# Morphological and molecular identification of *Cryptocotyle lingua* metacercariae isolated from Atlantic cod (*Gadus morhua*) from Danish seas and whiting (*Merlangius merlangus*) from the English Channel

**DOI:** 10.1007/s00436-021-07278-6

**Published:** 2021-08-27

**Authors:** Maureen Duflot, Mélanie Gay, Graziella Midelet, Per Walter Kania, Kurt Buchmann

**Affiliations:** 1 Laboratory for Food Safety, ANSES, Boulogne-sur-Mer, France; 2 University of Littoral Côte d’Opale, Boulogne-sur-Mer, France; 3 Laboratory of Aquatic Pathobiology, Department of Veterinary and Animal Sciences, Faculty of Health and Medical Sciences, University of Copenhagen, Frederiksberg C, Denmark

**Keywords:** *Cryptocotyle*, Morphological identification, Molecular identification, Heterophyidae, English Channel, Whiting

## Abstract

Trematode larvae (metacercariae) causing black spot disease occur frequently in gills, fins, skin and the superficial muscle layers of marine fish. Species within the genus *Cryptocotyle* Lühe, 1899 are frequently associated with this disease. Descriptions of the metacercarial stage are relatively limited and none has hitherto been reported from fish from the English Channel. The present study reports the morphological and molecular identifications of encysted black spot-inducing parasites from whiting (*Merlangius merlangus*) and Atlantic cod (*Gadus morhua*) caught respectively from the north coast of France (English Channel) and from Danish sea waters. Metacercariae were characterised morphologically based on microscopic observations and molecularly using Sanger sequencing of fragments of the mitochondrial *cox1* gene and rDNA *ITS* region. Morphological data were compared with available data in the literature. Phylogenetic trees including reference sequences were built to confirm morphological and molecular identifications. This survey constitutes the first description of *C. lingua* metacercariae in the English Channel ecosystems.

## Introduction

Marine fish are sometimes speckled with “black spots” induced by a host response to a trematode infection (Aalvik et al. 2015; Borges et al. 2015; Kristoffersen 1991; Sindermann & Farrin 1962). Several genera cause these symptoms. Among them, the genus *Cryptocotyle* Lühe, 1899 (synonyms *Tocotrema* Looss, 1899 and *Hallum* Wigdor, 1918 from Rees (1974)) is a taxon of trematodes belonging to the family Heterophyidae. According to the World Register of Marine Species (www.marinespecies.org), this genus includes 12 species, but only 6 of them circulate among inhabitants of marine and brackish waters. Most species have only a partial description of their host spectra. *Cryptocotyle badamshini* Kurochkin, 1959 was described only in marine waters and was first described in *Pusa caspica* from the Baltic Sea. *Cryptocotyle concava* Creplin 1825 was observed in freshwater snails, from the genus *Hydrobia*, and freshwater or brackish fish such as sticklebacks or gobies and has, as definitive hosts, birds such as gulls or ducks as well as cats (Gonchar 2020; Thieltges et al. 2008; Zander et al. 1984). *Cryptocotyle cryptocotyloides* Issaitschikow 1923 was first described as an avian parasite (Witenberg 1929). *Cryptocotyle delamurei* Jurachno 1987 was first described in the northern fur seal. *Cryptocotyle lingua* Creplin 1825 has *Littorina littorea* as the first intermediate host (Blakeslee et al. 2008; Landis et al. 2012), a variety of marine fish as second intermediate host such as gobies, gadoids, clupeids (Borges et al. 2015; Duflot, et al. 2021a; Goncharov et al. 2017) and piscivorous birds as final host (Linton 1915; Stunkard 1942). The first intermediate host of *Cryptocotyle jejuna* Nicoll, 1907 is *Hydrobia ulvae*, several species of gobies were described as second intermediate hosts and piscivorous birds as definitive host (Goncharov et al. 2017).

*Cryptocotyle* is a heteroxenous parasite (Smyth 1994; Stunkard 1929). Adults are present in the intestines of marine birds. Eggs are passed in bird host faeces and ingested by the first intermediate host, an aquatic snail. Eggs hatch and go through one free larval stage, the miracidium, and three successive larval stages in the snail: the sporocyst, the redia and the cercaria. Cercariae are released from the snail and actively swim to the next host, a fish, penetrating its skin and infecting the skin and the subcutaneous muscle of the fish host. It then encysts and develops into the metacercarial stage. The fish immune system responds with humoral and cellular reactions (Buchmann et al. 2009). Melanophores concentrate at the infection site; melanisation is visible to the naked eye (Mazzi 2004). Metacercariae protect their soft tegument against this fish immune response by developing a proteinaceous cyst wall resistant to pepsin and acids (El-Mayas & Kearn 2009). Birds belonging to the family Laridae, such as gulls, complete the *Cryptocotyle* life cycle by eating parasitised fish. Encysted metacercariae resist digestion by the bird’s gastric juices and migrate to the proximal intestine where they excyst in alkaline conditions when exposed to chymotrypsin and trypsin. Subsequently, the juvenile parasites attach to the avian intestinal wall and develop into the adult stage (Buchmann et al. 2009).

Some studies have described the distribution of *Cryptocotyle*. To date, it has been observed in Europe, North America and Asia. Host species reported infected with *Cryptocotyle* include *Littorina littorea* snails from Western Scotland (McQueen et al. 1973) and the Baltic, North and Celtic seas (Europe) (Thieltges et al. 2009), North American fish, i.e. herrings (*Clupea harengus*) from the northern New England coast (USA) (Sindermann & Farrin 1962) and cunners (*Tautogolabrus adspersus*) from the Newfoundland coast (Canada) (Sekhar 1970), as well as stray dogs from Rebun Island, Hokkaido (Japan) (Yoshimura 1965). Similarly, *Cryptocotyle* infections have been recorded in many North European marine fish species including Atlantic cod (*Gadus morhua*) (Borges et al. 2015; Koie 1984) and whiting (*Merlangius merlangus*) (Rea & Irwin 1991).

To our knowledge, no studies on *Cryptocotyle* infections in fish from the English Channel have been performed. Moreover, morphological measurements are mainly available for the adult stage (Linton 1915; Ransom 1920) and descriptions of metacercariae stages are still scarce (Borges et al. 2015; Linton 1915; Rees 1978). Over the past 15 years, molecular identification tools have been developed for *Cryptocotyle* (Blakeslee et al. 2008; Borges et al. 2015; Casalins et al. 2020; Duflot et al. 2021b; Gonchar 2020; Tatonova & Besprozvannykh 2019). In the present study, *Cryptocotyle* metacercariae sampled from naturally infected fish caught in the English Channel and the North Sea were morphologically described and molecularly characterised based on *cox1* gene and *ITS* region sequences.

## Materials and methods

### Sample collection

One batch of cod and one batch of whiting were fished and observed. Only parasitised fish were considered for the present study. Five parasitised Atlantic cod (*G. morhua*) individuals were caught by using net traps in Danish waters (Øresund) south of Helsingør, Denmark, in October 2019. Fish had a mean total length of 15.4 (14–16.5) cm. Six parasitised whiting (*M. merlangus*) were caught off the French coast of the English Channel between October and November 2019. Fish had a mean total length of 17.2 (15.7–17.9) cm.

### Parasite detection

To assess the level of infection, the location and number of black spots were observed and counted on each sample. Then, each fish was skinned and the subcutaneous muscle with a thickness of approximately 5 mm was removed from the fish. Skin and muscle samples were digested separately in a pepsin/HCl/saline solution (Borges et al. 2015) in 50-mL beakers under magnetic stirring (200 rpm, round magnet L = 40 mm) at 37 °C for 1 h. Digested samples were poured into Petri dishes. Metacercariae were collected by pipetting under an Olympus SZX16 stereomicroscope (Olympus Corporation, Tokyo, Japan). They were kept in phosphate-buffered saline (PBS) (pH = 7.3) at room temperature.

### Excystment of metacercariae

Metacercariae recovered from each sample were transferred to 30–1000 μL of 2% porcine trypsin (13,000–20,000 BAEE units/mg, Sigma-Aldrich, USA) in PBS (pH 7.3) at room temperature for no more than 2 h whereby the cyst wall opened and live metacercariae emerged. Excysted metacercariae were stored in 96% ethanol at 1 ± 1 °C.

### Morphological identification

Respectively, 5 and 10 excysted metacercariae from cod and whiting were analysed and identified under a light microscope (Leica DLMB 5000 B; Leica, Wetzlar, Germany). Excysted metacercariae stored in ethanol were rinsed in distilled water, stained with haematoxylin and mounted on slides in a glycerine gelatine medium (Buchmann 2007). The parasites and their main organs were measured (Table 1) (× 100–200 magnification) and photographed under a Leica DLMB microscope with a Leica DC300 camera. The parasites used for the measurements were uncompressed and parasites used for photos were flattened under a coverslip to bring as many morphological features into focus as possible.

**Table 1:** Morphometric data of metacercariae sampled from naturally infected whiting (*n* = 10) and from naturally infected cod (*n* = 5) and bibliographic data of metacercariae and adults. Measurements are expressed in mm. Numbers ① to ⑦ refer to Fig. 2; *n*, number of analysed individuals; *C.*, *Cryptocotyle*; *T.*, *Tocotrema*; *Mc*, Metacercariae

		Parasites in whiting (n = 10)		Parasites in cod (n = 5)		*C. lingua*	*C. lingua*	*C. lingua*	*T. lingua*	*C. concava*	*C. concava*	*C. jejuna*	*C. jejuna*	*C.* sp.
		Range	Average	Range	Average									
	Stage of maturity	Mc		Mc		Mc	Mc	Adult	Adult	Adult	Mc	Adult	Mc	Adult
	Body shape	Linguiform to pyriform		Linguiform to pyriform		-	Oval	Linguiform to pyriform	Linear to pyriform	Ovoid	Oval	Linguiform	Elongated	Linguiform
①	Distance from oral sucker to end of pharynx	0.07–0.12	0.10	0.08–0.11	0.09	0.03–0.04	-	0.03–0.05	-	0.06	0.049 (0.011 + 0.038)	0.04	0.026 (0.013 + 0.013)	-
②	Distance from oral sucker to intestinal branches	0.11–0.19	0.15	0.06–0.15	0.12	-	-	0.28–0.32	-	0.95	-	0.10	-	-
③	Width 1	0.11–0.31	0.23	0.20–0.26	0.23	0.18–0.21	0.19	0.20–0.90	0.35	0.85	0.37	0.23–0.6	-	0.37–0.45
④	Width 2	0.14–0.25	0.17	0.14–0.21	0.17	-	-	-	-	-	-	-	-	-
⑤	Oral sucker diameter	0.03–0.06	0.05	0.05–0.06	0.05	0.05–0.06	-	0.07–0.11	0.07	0.06–0.09	0.055	0.05	0.048	0.64–0.65
⑥	Ventrogenital complex diameter	0.02–0.05	0.03	0.02–0.03	0.02	0.02–0.03	-	0.12–0.25	-	0.15–0.30	-	0.06	-	-
⑦	Total length	0.44–0.92	0.60	0.39–0.59	0.51	0.58–0.68	0.48	0.55–2.00	0.75	1.00	0.42	1.80	-	0.72–0.90
	Reference	Present work				Borges et al. (2015)	Rees (1978)	Ransom (1920)	Linton (1915)	Ransom (1920)	Goncharov et al. (2017)	Ransom (1920)	Goncharov et al. (2017)	Zdzitowiecki et al. (1989)

### Molecular identification

Three excysted metacercariae per fish preserved in 96% ethanol were air-dried and lysed at 55 °C under orbital stirring at 300 rpm in 30 μL of lysis reagent (Borges et al. 2015) with a ThermoMixerC heater (Eppendorf, Hamburg, Germany). Complete lysis was confirmed by microscopy, whereafter proteinase K was inactivated by heating to 95 °C for 10 min (Thuy et al. 2010).

PCR reactions were performed in a total volume of 60 μL containing 2 μL of extracted genomic DNA, 1 unit of HotStarTaq DNA polymerase (Qiagen, Hilden, Germany), 1X reaction buffer, 0.1 mM dNTPs, 0.75 mM MgCl_2_ and 1 μM each primer (Eurofins, Nantes, France). A partial region of the mitochondrial *cox1* gene (approximately 350 bp) was amplified using primers JB3 (5’-TTT TTT GGG CAT CCT GAG GTT TAT-3’) and JB4.5 (5’-TAA AGA AAG AAC ATA ATG AAA ATG-3’) (Borges et al. 2015; Bowles et al. 1993). PCRs were carried out under the following conditions: 94 °C for 15 min (initial denaturation step), followed by 40 cycles of 94 °C for 1 min (denaturation), 50 °C for 1 min (annealing), 72 °C for 1 min (elongation) and a post-elongation step at 72 °C for 7 min, and then stored at 4 °C. The complete nucleotide sequence of the *ITS1-5.8S-ITS2* region of rDNA (approximately 1200 bp) was amplified using the primers BD1 (5’-GTC GTA ACA AGG TTT CCG TA-3’) and 28S1R (5’-AAG TAT TTA GCC TTG GAT GGA GTT T-3’) (Shumenko et al. 2017). PCRs were carried out under the following conditions: 94 °C for 15 min (initial denaturation step), followed by 35 cycles of 94 °C for 30 s (denaturation), 55 °C for 30 s (annealing), 72 °C for 2 min (elongation) and a post-elongation step at 72 °C for 5 min, and then stored at 4 °C. All PCR reactions were run on a Thermal Cycler (Applied Biosystems, Forster City, CA, USA).

All PCR products were run on a 2% agarose gel and stained with ethidium bromide for visualisation. PCR products of the expected size were sequenced twice and from both sides (forward and reverse), using Sanger sequencing (Genoscreen, Lille, France) with the above-described primers.

### Sequence alignment and phylogenetic analysis

Obtained sequences were visualised in BioEdit 7.0.9.0 software (Hall 1999); they were then assembled using MEGA 10.1.8 (Kumar et al. 2018). Ambiguous bases were clarified and called using the corresponding chromatograms. Nucleotide sequences were aligned using the Clustal W option of MEGA 10.1.8. A BLAST search was carried out for each DNA region (Altschul et al. 1997). Maximum Likelihood (ML), Neighbour Joining (NJ) and Minimum Evolution (ME) methods were conducted for separate and combined nucleotide data sets with other Heterophyidae sequences using MEGA 10.1.8. The trees were built with outgroups from the superfamily Opisthorchioidea (Table 2) and using 1000 bootstrap replications. Similar trees were observed, ML trees giving higher bootstrap values are shown. The most suitable fit model of each gene fragment was determined using the corrected Akaike Information Criterion (AICc) and the Bayesian Information Criterion (BIC) on the 24 models tested in MEGA 10.1.8. *ITS* and *cox1* sequences were respectively fitted to the JC (+ I) model (Jukes & Cantor 1969) and the Tamura-Nei model (Tamura & Nei 1993). The highest log likelihoods were respectively (− 3406.77) and (− 7906.05).

**Table 2:** Molecular sequences investigated in the present study

Species	Reference	GenBank accession	
		*cox1*	*ITS*
*Apophallus donicus*	Sándor et al. (2017)	MF438082	MF438057
*Apophallus muehlingi*	Sándor et al. (2017)	MF438078	MF438068
*Apophallus* sp.	Ferguson et al. (2012)	JQ241159	-
*Cryptocotyle dominicana*	Casalins et al. (2020)	MG717397-MG717399; KY968654-KY968656	MK239018-MK239021
*Cryptocotyle lingua*	Borges et al. (2015)	KJ711861-KJ711866	KJ641518-KJ641524
	Blakeslee et al. (2008)	EU876512-13; EU876492; EU876363; EU876390; EU876416; EU876420; EU876429	-
	This study	MW542531-MW542559	MW544112-MW544139
*Cryptocotyle lata*	Tatonova and Besprozvannykh (2019)	-	MH025622-MH025623
*Galactosomum* sp.	Leung et al. (2009)	FJ765489	-
*Haplorchis pumilio*	Lopes et al. (2020)	MT831076	MT840093
*Haplorchis taichui*	Lee et al. (2013)	KF214770	-
	Dao et al. (2017)	MF287788	-
*Heterophyes heterophyes*	Henedi (2019)	-	KX431325
*Heterophyes* sp.	Masala et al. (2016)	-	KU674954
*Metagonimus suifunensis*	Shumenko et al. (2017)	-	KX387514
*Procerovum* sp.	Arya et al. (2016)	-	KM226892
*Pygidiopsis summa*	Lee et al. (2004)	AF181884	-
*Stellantchasmus falcatus*	Chontananarth et al. (2014)	KF044301	-
*Stellantchasmus* sp.	Wongsawad et al. (2017)	-	KU753591
**Outgroups**			
*Clonorchis sinensis*	Lee and Huh (2004)	AF181889	-
	Dao et al. (2017) Tatonova et al. (2012)	MF287785 -	- JQ048601
*Opisthorchis viverrini*	Thaenkham et al. (2011)	HQ328544	-
	Dao et al. (2017)	MF287782	-
	Sanpool et al. (2018)	-	MG797539
*Opisthorchis sudarikovi*	Suleman et al. (2019)	-	MK227161

## Results

**Fig. 1:**
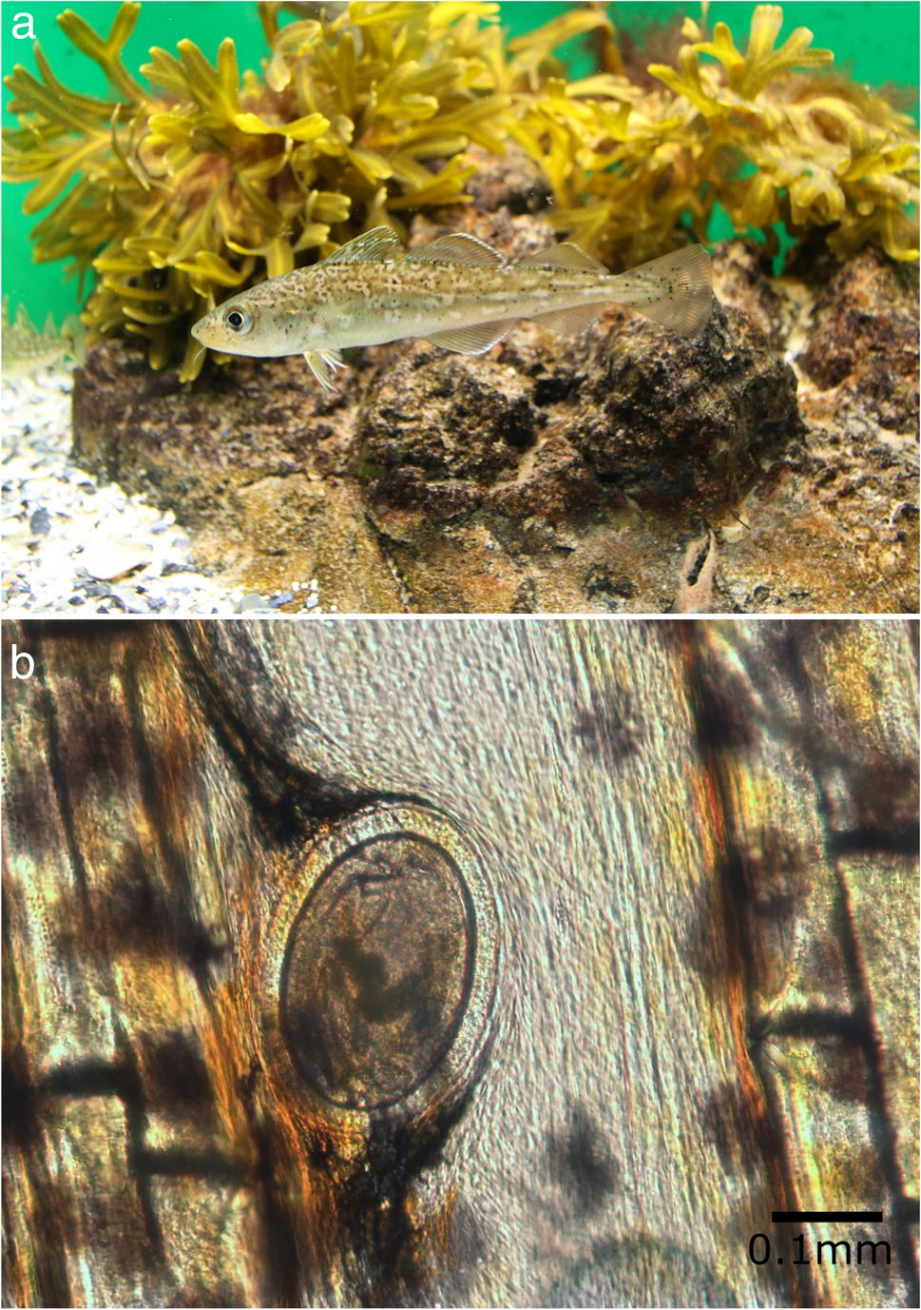
Infected Atlantic cod (*Gadus morhua*) (**a**) and microscope observation of encysted metacercariae in caudal fin (**b**)

The intensity of infection varied between 120 and 173 black spots per fish for the five analysed cod samples (Fig. 1 a). The intensity varied between 10 and 349 black spots per fish for the six analysed whiting samples. Metacercariae were mainly localised on the fins, skin, subcutaneous muscles and especially in paraspinal connective tissues in both fish species (Fig. 1 b).

### Morphological identification

**Fig. 2:**
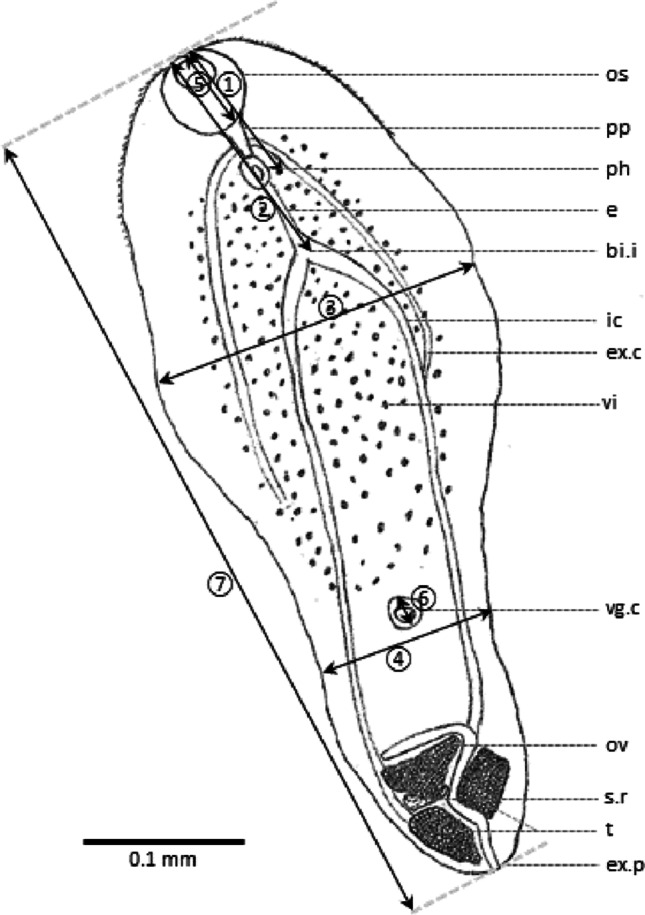
Morphology of excysted *Cryptocotyle lingua* metacercariae (ventral view) from *Gadus morhua* and *Merlangius merlangius*. Abbreviations: bi.i, bifurcation of intestine; e, oesophagus; ex.c, excretory canal; ex.p, excretory pore; ic, intestinal caecum; pp, prepharynx; ph, pharynx; ov, ovary; os, oral sucker; s.r, seminal receptacle; t, testis; vg.c, ventrogenital complex; vi, vitellaria; ① distance from oral sucker to end of pharynx; ② distance from oral sucker to intestinal branches; ③ width 1; ④ width 2; ⑤ oral sucker diameter; ⑥ ventrogenital complex diameter; ⑦ total length

Classical measurements were taken for 15 metacercariae (Table 1). Based on observations, the general morphology was drawn (Fig. 2).

### Description of metacercariae from whiting (based on 10 whole-mounted specimens)

**Fig. 3:**
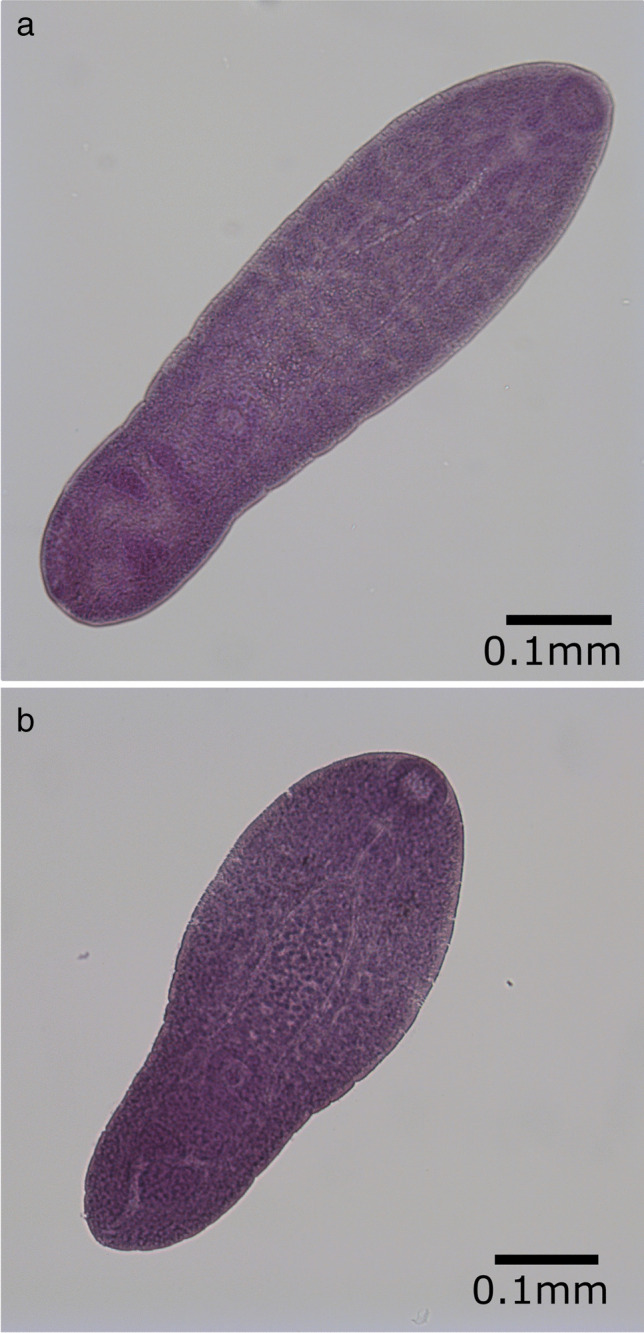
Excysted *Cryptocotyle lingua* metacercariae at different degrees of contraction **a** in whiting from the English Channel, **b** in cod from Danish waters

Excysted metacercariae linguiform to pyriform according to the state of contraction at fixation in ethanol (Fig. 3 a), length 0.60 (0.44–0.92) mm, first width 0.23 (0.11–0.31) mm, maximum wide or second width at the posterior end of body 0.17 (0.14–0.25) mm; anterior body covered by scale-like spines; oral sucker subterminal with a circular aperture, 0.05 (0.03–0.06) mm in diameter; prepharynx short and pharynx elliptical; oesophagus rectilinear; intestinal bifurcation occurs at one-fourth of body length; ventral sucker 0.03 (0.02–0.05) mm in diameter, on median line from one-half to two-thirds of the total length according to the condition of contraction; testes immature, in the posterior end of the body, detected close to the median line; structures assumed to be seminal receptacles detected in some metacercariae, ovary just pre-testicular in the posterior end of the body.

### Description of metacercariae from cod (based on five whole-mounted specimens)

Body shape linguiform to pyriform according to the state of contraction (Fig. 3 b), length 0.51 (0.39–0.59) mm, maximum width in anterior end 0.23 (0.20–0.26) mm and a second width in posterior end 0.17 (0.14–0.21) mm; tegument slightly spine anteriorly; oral sucker subterminal and round, 0.05 (0.05–0.06) mm in diameter; prepharynx short and pharynx elliptical; oesophagus straight; intestinal bifurcation occurs at one-fourth of body length; ventral sucker small, 0.02 (0.02–0.03) mm in diameter, on median line at one-half to two-thirds of the total length according to the condition of contraction; testes immature, in the posterior end of the body, detected close to the median line; structures assumed to be seminal receptacles detected in some metacercariae, ovary just pre-testicular in the posterior end of the body.

### Molecular identification and phylogenetic data

PCR on the *cox1* gene of mtDNA was performed on 29 parasites: 15 isolates from infected whiting and 14 isolates from infected Atlantic cod. PCR products were about 320 bp long. Likewise, PCR of the *ITS* region of rDNA from 28 parasites including 10 isolates from whiting and 18 isolates from cod resulted in a product of about 1700 bp. Sequences of the *cox1* gene and *ITS* region were deposited in GenBank under the accession numbers MW542531-MW542559 and MW544112-MW544139, respectively. BLAST analysis of the *cox1* fragments (281 bp) led to 100% of similarity with GenBank accession nos. KJ711861, KJ711862, KJ711865, KJ711866 (*C. lingua*, Baltic Sea, Denmark) and EU876519 (*C. lingua*, Canada) for 24 sequences. Four sequences had 99.69% similarity with the KJ711866 sequence, each at a different position (G^9^ → A^59^; T^79^ → C^129^; G^215^ → C^265^; G^276^ → A^326^ (Query → KJ711866)). The last sequence differed from the KJ711866 sequence at two positions (T^79^ → C^129^; C^105^ → T^154^) and showed 99.38% similarity. All *ITS* sequences (1708 bp) showed 98 to 100% similarity with GenBank accession nos. KJ641518 to KJ641524 (*C. lingua*, Baltic Sea, Denmark). In conclusion, all analysed sequences showed high similarities with *C. lingua* sequences.

**Fig. 4:**
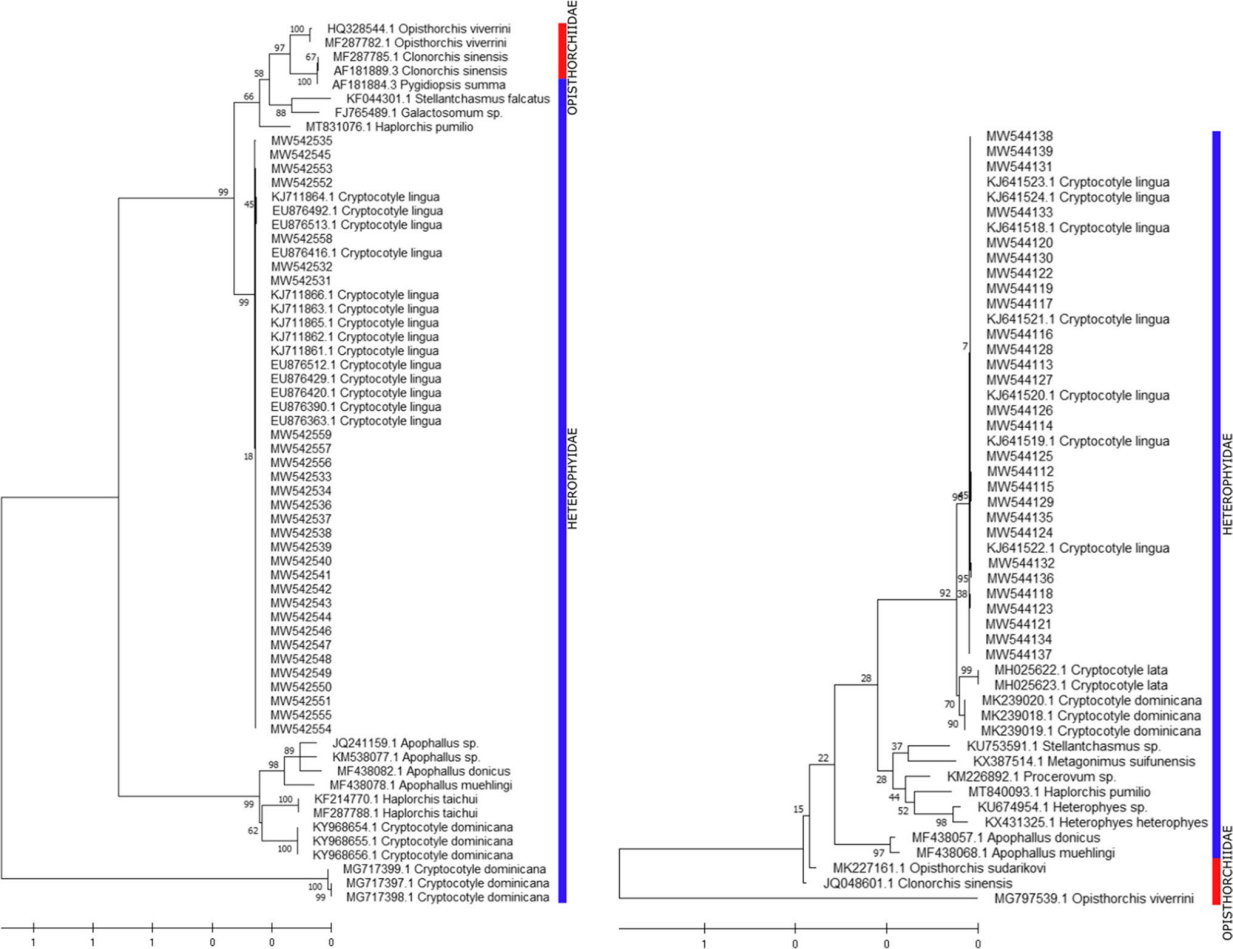
Phylogenetic trees based on *cox1* mtDNA (left tree) and *ITS* rDNA (right tree) sequences using the ML method with 1000 bootstraps

Trees based on nucleotide sequences of the *cox1* gene (281 bp) and the *ITS* region (1708 bp) showed that all sequences clustered with *C. lingua* with high bootstrap values (Fig. 4) and confirmed BLAST results. In addition, all *C. lingua* sequences, isolated from parasites sampled in the Baltic Sea, Denmark, North America or Europe, form a monophyletic clade.

## Discussion

In this study, parasites inducing black spots on the skin of whiting and cod from northern Europe were identified. Characteristic black melanisation is due to the concentration of melanophores at parasite infection sites (Zander et al. 1984). Several heterophyid genera are responsible for this pathology, e.g. *Apophallus* (Sándor et al. 2017), *Cryptocotyle* (Chapman & Hunter 1954), *Haplorchis* (Paperna & Dzikowski 2006) and *Stellantchasmus* (Chai et al. 2016).

The metacercariae presented in this study belong to *Cryptocotyle* according to their morphological traits and organ sizes, and their molecular identification. The literature holds only a few accurate descriptions of metacercariae from this genus with measurements of the main characteristic organs. Due to this scarcity, the data of this study were compared with published descriptions of metacercariae (Borges et al. 2015; Goncharov et al. 2017; Rees 1978) and adults (Ransom 1920; Zdzitowiecki et al. 1989). Linton (1915) compared metacercariae from the skin of fish and used adult stages from the intestine of loons as a reference for identification. In most studies, morphological identification of trematode larval stages in fish requires experimental infections to grow the parasite to its adult stage. The adult stage is the only trematode stage in which all the organs are present. However, different stages of maturity (with different degrees of organ development) can be observed in the final host’s intestinal tract (McCarthy & Hassett 1993). In their most suitable hosts, the parasites can grow to a large size and can produce many eggs (Witenberg 1929); in other hosts, the development is not complete, and size can vary (Yoshimura 1965). For example, observing *C. lingua* in domestic rats, cats and ducks, Stunkard (1929) found that worms that had developed in domestic cats were larger than those recovered from rats, and reproductive organs were not well developed and few eggs were present. He concluded that *C. lingua* could not mature in domestic ducks. Morphological characteristics thus appear to be host dependent, and each developmental stage needs to be compared with equivalent reference individuals for reliable identification. Here, all the metacercariae from *G. morhua* and *M. merlangus* displayed the expected organs of young *Cryptocotyle* trematodes of marine species. The appearance and organ positions were similar in specimens from both fish species studied (Table 1). The studied specimens showed the most morphometric similarities with *C. lingua* metacercariae described in Borges et al. (2015) and Rees (1978). *Cryptocotyle lingua* differs from *C. concava* and *C. jejuna* by its shape and smaller width according to Ransom (1920). However, *C. lingua* metacercariae from the present work and former description from Borges et al. (2015) and Rees (1978) had a total length in the same range as metacercariae from *C. concava* described by Goncharov et al. (2017), whereas their width was considerably smaller. Moreover, regarding total length, parasites differed to *C. concava*, *C. jejuna* and *C.* sp. descriptions by Ransom (1920) and Zdzitowiecki et al. (1989). The distance from the oral sucker to the end of the pharynx was bigger than the ones described by Borges et al. (2015) and Goncharov et al. (2017) for *C. lingua*, *C. concava* and *C. jejuna* metacercariae. The metacercariae studied in this work were markedly smaller than the sizes reported for *C. lingua* adult stages. The measurements were close to minimum values for width, oral sucker diameter, distance from oral sucker to intestinal branches and total length. Therefore, the parasites sampled from whiting and cod matched the descriptions and measurements of *C. lingua* adults observed by Linton (1915), Ransom (1920), Stunkard (1929), Witenberg (1929) and Yoshimura (1965) and the descriptions of metacercariae in Borges et al. (2015) and Rees (1978). These observations are consistent with Pearson’s conclusions, cited by Williams and Jones (1994), who considered metacercariae as a juvenile resting stage before adulthood, infecting the definitive host in which it reaches sexual maturity.

Experimental infection of final hosts to study the growth and development of intermediate-stage parasites is laborious and requires laboratory animals. Moreover, specific determination of larval stages based on morphological traits remains difficult, subjective and ambiguous due to the lack of reproductive system traits. Molecular identification to confirm the morphological identification of isolates is strongly recommended. Few sequences are available for the *Cryptocotyle* genus in databases (Blakeslee et al. 2008; Borges et al. 2015; Casalins et al. 2020; Duflot et al. 2021b; Tatonova & Besprozvannykh 2019). In this study, *ITS* and *cox1* DNA sequences of *C. lingua* from Atlantic cod and whiting were added to the GenBank database. On the *cox1* gene, no significant similarity was found with the available sequences of the other parasites causing black spot diseases (*Apophallus*, *Haplorchis*, *Stellantchasmus*). Likewise, the percentage of similarity (4 to 96%) and of query cover (10 to 70%) was lower for other parasites inducing black spot disease than for *Cryptocotyle*. Phylogenetic trees on the *cox1* gene and *ITS* region showed differences for *C. dominicana* and outgroup phylogenetic positions. This result can be attributed to the evolutionary history of these selected regions. For instance, the selected *cox1* region is a short fragment compared with the *ITS* region sequence. In both trees, phylogenetic comparisons within the *Cryptocotyle* genus revealed high sequence similarity for both parasite origins with *C. lingua* cercariae and metacercariae sampled in North America and Europe (Spain and Norway coasts) (Blakeslee et al. 2008) and in the Baltic Sea (Borges et al. 2015), respectively. On the phylogenetic tree based on *ITS* region sequences, *C. lingua*, *C. lata* and *C. dominicana* were included in a monophyletic group, whereas other Heterophyidae organisms (*Stellantchasmus*, *Paragonimus*, *Procevorum*, *Haplorchis* and *Heterophyes*) were gathered in another monophyletic group. However, both these monophyletic groups seemed to share a common ancestor.

In Scandinavian waters, digenetic trematode infections have been reported in Atlantic cod, with *C. lingua* metacercariae being observed in samples from off the coast of Norway (Heuch et al. 2011) and in 11 of the 15 sampling stations in Danish and adjacent waters (Koie 1984). No previous investigations have been carried out on fish from the English Channel geographic area. Thus, the findings of *C. lingua* in *G. morhua* from Danish seas are consistent with previous studies and confirm the presence of *C. lingua* cercariae in *L. littorea* snails in the Baltic, Celtic and North seas (Thieltges et al. 2009) and in some marine samples from England (Rothschild 1942).

In summary, this paper described for the first time the presence of *C. lingua* in the skin and subcutaneous muscle of *M. merlangus* from the English Channel using morphological and molecular tools and further confirms the presence of *C. lingua* in *G. morhua* in Baltic waters (Borges et al. 2015; Koie 1984). The comparison of morphological and molecular methods needs to be developed in parasitological research, especially for trematode parasites, because molecular data lack for many trematode genera and species. The recent development of molecular tools will help the detection and observation of trematodes at different developmental stages. This complementary approach, based on morphological and molecular data, should prove valuable for poorly described and characterised trematodes and for epidemiological studies on the distribution of *Cryptocotyle* species in different geographical areas, under different environmental conditions and in a wide variety of fish species.

## Data Availability

Not applicable.
